# Iron chelation effect of curcumin and baicalein on aplastic anemia mouse model with iron overload

**DOI:** 10.22038/ijbms.2019.30840.7440

**Published:** 2019-06

**Authors:** Wu Dijiong, Wen Xiaowen, Xu Linlong, Liu Wenbin, Hu Huijin, Ye Baodong, Zhou Yuhong

**Affiliations:** 1 Department of Hematology, First Affiliated Hospital of Zhejiang Chinese Medical University, Hangzhou, Zhejiang, China; 2 Department of Internal Medicine, Central Hospital of Jinhua Affiliated to Zhejiang University, Jinhua, Zhejiang, China; 3 Department of Hematology, Taizhou Central Hospital, Taizhou, Zhejiang, China

**Keywords:** Anemia, Animal, Aplastic, Baicalein, Curcumin, Deferoxamine, Iron overload, Mice, Models

## Abstract

**Objective(s):**

The current work aimed to assess whether curcumin and baicalein can chelate iron in aplastic anemia (AA) complicated with iron overload, exploring the potential mechanisms.

**Materials and Methods:**

A mouse model of AA with iron overload complication was firstly established. Low and high-dose curcumin or baicalein treatment groups were set up, as well as the deferoxamine positive control, normal and model groups (n=8). Hemogram and bone marrow mononuclear cell detection were performed, and TUNEL and immunohistochemical staining were used to evaluate hematopoiesis and apoptosis in the marrow. ELISA, Western blot, and qRT-PCR were employed to assess serum iron (SI), serum ferritin (SF), bone morphogenetic protein 6 (BMP-6), SMAD family member4 (SMAD4) and transferrin receptor 2 (TfR2) amounts.

**Results:**

Both curcumin and baicalein improved white blood cell (increase of 0.28-0.64×10^9^/l in high-dose groups) and hemoglobin (increase of around 10 g/l) amounts significantly, which may related to decreased apoptosis (nearly 30%-50% of that in the model group) in the bone marrow, while their effects on platelet recovery were limited and inferior to that of deferoxamine (DFO). Both test compounds up-regulated hepcidin and its regulators (BMP-6, SMAD, and TfR2) at the protein and mRNA levels; high dosage treatment may be beneficial, being better than DFO administration in lessening iron deposition in the bone marrow.

**Conclusion:**

Curcumin and baicalein protect hematopoiesis from immune and iron overload-induced apoptosis, exerting iron chelation effects *in vivo.*

## Introduction

Aplastic anemia (AA) represents an ailment of bone marrow deficiency featuring pancytopenia, with a high risk of infection and hemorrhage (1). During the course of AA, several factors, including inappropriate use of drugs with potential bone marrow depression risk, microbial infection, and iron overload, may aggravate this failure (2-4). Owing to dysfunctional iron metabolism and excessive transfusion, iron overload is one of the most commonly encountered complications of AA, especially chronic AA (5), which was speculated to be detrimental to hematopoiesis and the immune system (6, 7). 

Multiple clinical iron chelators are available, including deferasirox (DFX) (8) and deferoxamine (DFO) (9), with tolerability and satisfactory efficiency in decreasing the serum and organ iron burdens. Iron chelation attracts increasing attention due to its benefit in protecting liver and heart functions, while promoting hematopoiesis, which all benefit life quality improvement (10). However, these drugs have some drawbacks. DFO needs to be continuously administered subcutaneously for hours every day, which makes it inconvenient for outpatients; DFX is an oral agent with improved patient compliance, but cost-inefficient for some patients (11). Therefore, developing and identifying effective and low cost iron chelation agents is clinically essential.

Baicalein and curcumin are active components from Scutellaria and Zingiberaceae (such as curcuma and turmeric), which are effective against tumors, infections, and oxidative stress disorders (12-15). These components are widely used in China (16, 17). Recently, curcumin was shown to affect iron accumulation and oxidative stress in a rat model of chronic iron overload, thereby impairing iron absorption (18, 19). On the other hand, baicalein also shows iron-binding characteristics and can form an iron-baicalein complex (20, 21), indicating its potential effect on iron chelation. The protective effects of these compounds in AA complicated with iron overload, and whether they can benefit hematopoietic recovery are largely unknown. In this study, we attempted to address these concerns in model mice with AA complicated with iron overload (22) in order to explore the potential mechanisms.

## Materials and Methods

***Animals***

A total of 56 specific-pathogen-free (SPF) BALB/c mice (female, 6–8 weeks of age), alongside five DBA/2 mice (female, 6–14 weeks of age) were obtained from the Laboratory Animal Center of Zhejiang Chinese Medical University. The study had approval from the Animal Management and Ethics Committee of Zhejiang Chinese Medical University (NO. ZSLL-2013-108) and carried out according to the National Institute of Health Guide for the Care and Use of Laboratory Animals (21).

***Reagents***

Iron dextran (cat: D8517, Sigma, USA); curcumin (cat: L4352, Nanjing Zelang Chinese Medicine Science and Technology Co Ltd); baicalein (cat: L8562, Nanjing Zelang Chinese Medicine Science and Technology Co Ltd); deferoxamine pure powder (Novartis Pharma Schweiz AG, Switzerland); serum iron detection kit (cat. A039-1, Nanjing Jiancheng Bioengineering Institute, China); serum ferritin (SF) ELISA kit (cat. CSB-E05187h, Cusabio Biotech Co, Ltd, China); TUNEL apoptosis assay kit (cat. S7100, Millipore, USA); rabbit anti-Bax (cat: 50599-2-Ig), anti-caspase 3 (cat: 19677-1-AP), anti-caspase 9 (cat: 10380-1-AP) and anti-RARP1 (cat: 13371-1-AP) polyclonal antibodies  (Proteintech Group, Inc, USA); anti-Bcl-2 mouse monoclonal antibodies (cat: sc-7382, Santa Cruz Biotechnology, Inc, USA).

***Animal grouping***

BALB/c mice were assigned to five groups, including the normal control (Normal, n=8), DFO-treatment (DFO, n=8), low-dose curcumin-treatment (Cur (low), n=8), high-dose curcumin-treatment (Cur (high), n=8), Model control (Group D, n=8), low-dose baicalein-treatment (Bai (low), n=8) and high-dose curcumin-treatment (Bai (high), n=8) groups.

***Establishment of the mouse model***

Mouse model establishment was conducted as previously described by our team (22). In brief, the animals were firstly intraperitoneally injected with 200 mg/kg/week iron dextran (10 weeks). Then, all animals underwent whole-body radiation (^60^Co 6.0 Gy, 1 Gy/min). Within 4 hr, 0.2 ml thymic cell suspensions (5×10^6^ cells/ml) from DBA/2 mice were infused through the tail vein. 

***Animal treatments***

DFO at 0.2 g/kg (solubilized in saline) was subcutaneously injected twice a day in the abdominal area for 5 weeks; curcumin (low and high doses of 1 and 4 g/kg, respectively) and baicalein (low and high doses of 0.6 and 2 g/kg, respectively) were dissolved in corn oil and administered intragastrically once a day for 5 weeks. Control (Normal and Model) groups were administered similar volumes of subcutaneous saline and intragastric corn oil, respectively. All treatments started from thymic cell injection.

***Sample collection***

The day of thymic cell injection was considered day 0. On days 7, 14 and 35, peripheral blood samples were obtained by the retro-orbital route for routine blood test. On day 35, blood, liver, and bilateral femur specimens were collected from random animals (n=4) after overnight fasting. 

***Complete blood count***

Manual classification and counting were performed to detect the peripheral hemogram (23). Briefly, blood smear preparation was carried out for Wright-Giemsa staining, after which a technician analyzed the samples by light microscopy at ×400 (CX31RTSF, Olympus Optical Co, Ltd, Japan).

***Pathomorphology and iron deposition assessments***

After sample collection, liver and unilateral femur samples were firstly submitted to fixation with 10% formalin (24 hr); femur specimens underwent further decalcification with 5% nitric acid (7-12 hr). Following dehydration, paraffin-embedding, sectioning, and staining by hematoxylin and eosin (H&E)/iron (Sudan red), the pathomorphological features of the bone marrow as well as iron accumulation in many organs were observed by light microscopy at ×400.

***SI level assessment***

Plasma SI amounts were assessed spectrophotometrically on a microplate reader, and derived as follows: 

SI =$\frac{\mathrm{Ad}-\mathrm{Ab}}{\mathrm{As}-\mathrm{Ab}}$ × standard concentration (35.81μmol/l)

Where Ad, Ab and As are absorbance values of the detection, blank tube and standard’s tubes, respectively.

**Table 1:** Primers for Hepcidin, BMP6, SMAD4, TfR2, and GAPDH detection

Gene	Direction	Sequence (5′-3′)	Length (bp)
Hepcidin	F	CTGAGCAGCACCACCTATCTC	205
	R	TGGCTCTAGGCTATGTTTTGC	
BMP6	F	ATGGCAGGACTGGATCATTGC	54
	R	CCATCACAGTAGTTGGCAGCG	
SMAD4	F	AGGTGGCCTGATCTACACAAG	111
	R	ACCCGCTCATAGTGATATGGATT	
TfR2	F	ATTCTCCTTTCTCCCTCTTT	253
	R	GCTGTCCATCTCACTCTCTA	
GAPDH	F	CCTCAAGATTGTCAGCAAT	141
	R	CCATCCACAGTCTTCTGAGT	

BMP6, bone morphogenic protein 6; SMAD 4, SMAD family member 4; TfR2, transferrin receptor 2; F, forward; R, reverse.

**Table 2:** Levels of Bone Marrow Cell Apoptosis Related Proteins [M(QR)]

	Bax	Bcl-2	Caspase-3	Caspase-9	RARP
Normal	4(2)	6(3)	2(2)	2(2)	4(0)
DFO	6(2)	6(3)	4(0) +	4(2)	4(2)
Curcumin (low)	6(0)*	9(3)	4(2)	6(0)*	4(2)
Curcumin (high)	4(2)	6(0)	2(2) +	4(2)	4(2)
Model	6(3)*	6(2)	6(3)*	6(3) *	6(3)*
Baicalein (low)	6(2)	6(0)	4(0) +	6(3) *	6(0)*
Baicalein (high)	4(2)	9(3)	4(0)+	4(2)	4(2)

Immunohistochemical staining of bax, bcl-2, caspase-3, caspase-9 and RARP in different groups was performed. Staining intensity and the percent of positive cells were scored. Data are presented as median (interquartile range),

* P<0.01 (compared with the Normal group),

+ P<0.05 (compared with the Model group).

***ELISA for SF detection***

Serum (0.5 ml) was used to detect SF amounts with a specific ELISA kit, as directed by the manufacturer. 

***TdT-mediated dUTP-biotin nick end labeling assay (TUNEL) assay***

After fixation with 10% formalin for 24 hr, femur specimens were further decalcified with 5% nitric acid (7-12 hr). After dehydration, paraffin-embedding and sectioning (4 μm), HRP-conjugated dUTP was added for incubation. The TUNEL apoptosis assay kit was employed as directed by the manufacturer. A fluorescence microscope (Olympus BX51T-PHD-J11, Japan) was employed to count positive nuclei in various samples.

***Immunohistochemistry (IHC) for the detection of apoptosis-related proteins in the bone marrow***

Staining intensity was assessed as “–” (no staining), “+” (mild staining, with positive cells showing yellow signals), “++” (moderate staining, with positive cells displaying brown signals) and “+++” (intense staining, with positive cells showing dark-brown signals), with scores of 0, 1, 2 and 3, respectively. Staining rate was determined as (–) (no stained cells), (+) (stained cells <25% of all cells), (++) (stained cells comprising 26-50%) and (+++) (stained cells >50%), with scores of 0, 1, 2 and 3 points respectively. Both subscores were multiplied to obtain the final score (24). 

***Western blot analysis***

Cell lysis was performed with chilled lysis buffer (10 mM Tris pH 7.5, 130 mM NaCl, 1% NP-40, 10 mM NaPPi, 1 mM PMSF, 0.1 mM Na_3_VaO_4_). The resulting lysates were centrifuged at 11900 g (15 min, 4 ^°^C). The Bradford reagent was employed for protein quantitation as directed by the manufacturer. β-actin served as a loading control. Equal amounts of total protein were resolved by 12% SDS-PAGE and electro-transferred onto polyvinylidene difluoride (PVDF) membranes (Millipore, USA). Primary and horseradish peroxidase (HRP)-linked secondary antibodies were successively incubated with the samples. ECL reagents (Beyotime, China) and X-ray film (Kodak, Japan) exposure were used for detection and development, respectively. The Image J 1.46r software was employed for analysis. Anti-hepcidin, anti-BMP6, anti-SMAD4, anti-TfR2 (Abcam, USA), and anti-β-actin (LiankeBio, China) monoclonal antibodies were probed.

***RT-qPCR***

Total RNA extraction from hepatocytes was performed with TRIzol (Invitrogen; USA). RT was carried out with a PrimeScript™ RT reagent kit with gDNA Eraser (Takara Biotechnology Co, Ltd, China) as instructed by the manufacturer, with 1 μg total RNA. First strand cDNA was obtained with PrimeScript 1^st^ strand cDNA Synthesis Kit (Takara Biotechnology) as instructed by the manufacturer. SYBR^®^ Premix Ex TagTM (Takara Biotechnology) was employed for qPCR in 20 μl-reactions (2 μl cDNA, 10 μl SYBR^®^ Premix Ex TagTM (2×), 0.8 μl of each primer, 0.4 μl ROX Reference Dye and 6 μl nuclease-free water). Amplification was performed at 95 ˚C for 30 sec, followed by 40 cycles of 95 ˚C (5 sec) and 60 ˚C (30 sec); 95 ˚C (15 sec), 60 ˚C (30 sec) and 95 ˚C (15 sec) was used for separation. The ΔCq values (Cq _target gene_ – Cq _reference gene_) for various samples were derived by the 2^-ΔΔCq ^method: ΔΔCq=ΔCq _experimental group_ - ΔCq _normal control group_(25). GAPDH was employed for normalization; primers are listed in Table 1. 

***Statistical analysis***

SPSS 17.0 was used for all analyses. Normally distributed parameters were presented as mean±standard deviation (SD), and those with skewed distribution as median and interquartile range. Analysis of variance (ANOVA) and pairwise comparisons were employed for normally distributed data; the Kruskal–Wallis H and Mann–Whitney U test were employed for pairwise and multiple comparisons of non-normally distributed data, respectively. *P<*0.05 indicated a statistically significant difference.

## Results

***Hemogram and bone marrow mononuclear cells***

**Figure 1:**
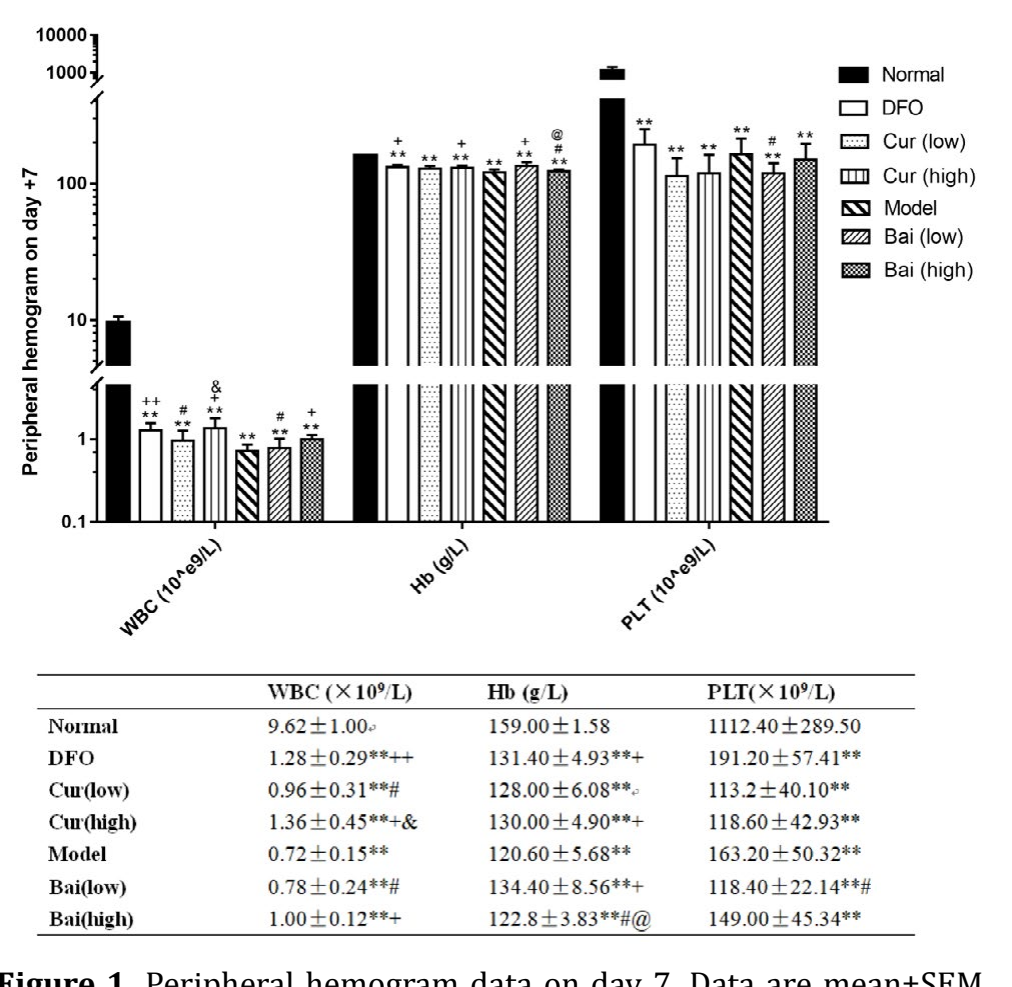
Peripheral hemogram data on day 7. Data are mean±SEM (n=8), **P<0.01 (vs Normal group); +*P<*0.05, ++*P<*0.01 (vs Model group); #*P<*0.05 (vs DFO group); @*P<*0.05 (vs corresponding low-dose group); & *P<*0.05 (vs low-dose baicalein)

WBC: white blood count; Hb: hemoglobin; PLT: platelet; DFO: Deferoxamine; Cur: Curcumin; Bai: Baicalein

**Figure 2:**
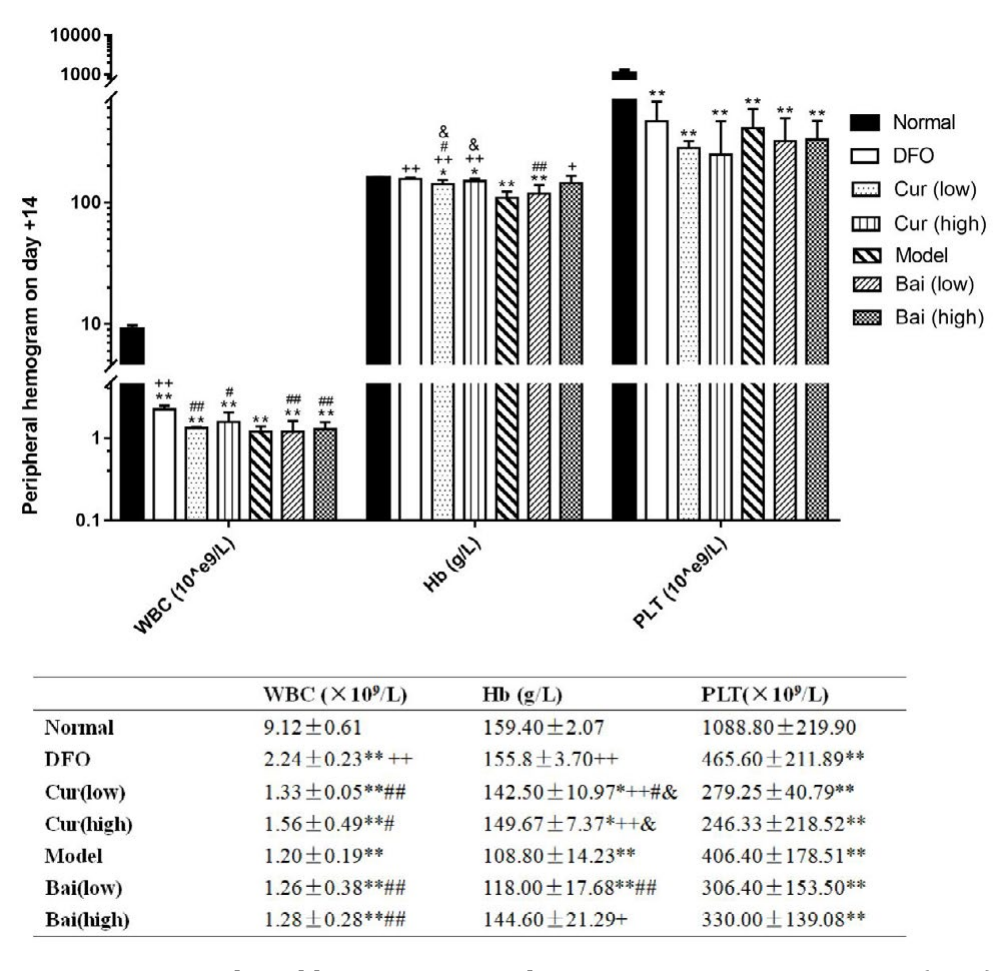
Peripheral hemogram on day 14. Data are mean±SEM (n=8), *P<0.05, **P<0.01 (vs Normal group); ++*P<*0.01 (vs Model group); #*P<*0.05, ##*P<*0.01 (vs DFO group); & *P<*0.05 (vs low-dose baicalein)

WBC: white blood count; Hb: hemoglobin; PLT: platelet; DFO: Deferoxamine; Cur: Curcumin; Bai: Baicalein

**Figure 3:**
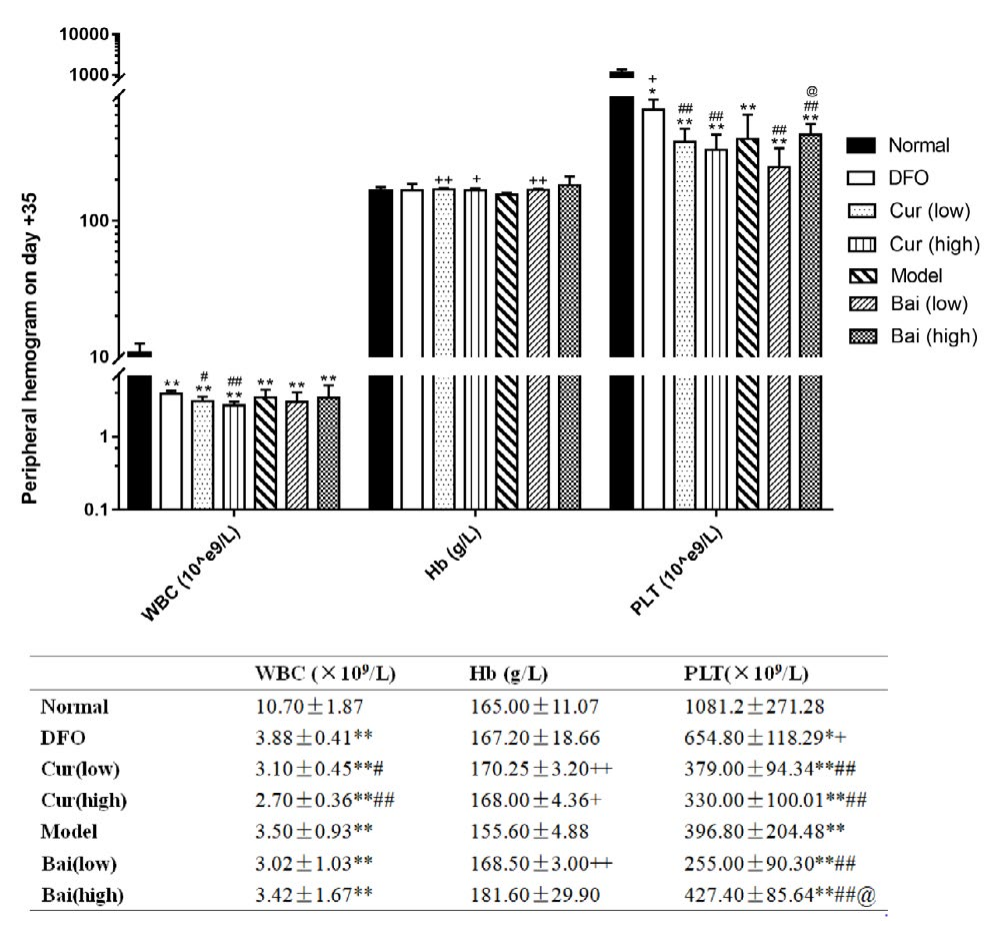
Peripheral hemogram on day 35. Data are mean±SEM (n=8), *P<0.05, **P<0.01 (vs. Normal group); +*P<*0.05, ++*P<*0.01 (vs. Model group); #*P<*0.05, ##*P<*0.01 (vs. DFO group); @*P<*0.05 (vs. corresponding low-dose group)

WBC: white blood count; Hb: hemoglobin; PLT: platelet; DFO: Deferoxamine; Cur: Curcumin; Bai: Baicalein

Hemogram was performed on days 7, 14, and 35 to verify the AA mouse model and assess the recovery of hematopoiesis. On day 7 (Figure 1), all model groups showed significantly decreased white blood cell (WBC), hemoglobin (Hb), and platelet (PLT) levels compared with the normal group (mean WBC, 9.62×10^9^/l; Hb, 159 g/l; PLT, 1112.4×10^9^/l). Notably, treatment with DFO and high-dose curcumin and baicalein resulted in increased WBC amounts (means of 1.28, 1.36 and 1×10^9^/l, respectively, vs 0.72×10^9^/l in the model group, all *P*<0.05); DFO distinctly affected WBC levels compared with both test drugs at low-dose (means of 0.96 and 0.78×10^9^/l, respectively; *P*<0.05 and *P*<0.01, respectively). Moreover, DFO, high-dosage curcumin, and low-dosage baicalein significantly increased Hb levels equivalently (means of 131, 130 and 134.4 g/l, respectively vs 120.6 g/l in untreated model animals, *P*<0.05). No effects were observed on PLT recovery for all treatments. Hb levels in the high-dose curcumin group were lower compared with those of the low-dose group (*P*<0.05). WBC amounts after treatment with high-dose curcumin were higher than those of the low-dose baicalein group. On day 14 (Figure 2), the Model control group had lower levels of Hb than on 7d, although not statistically significant. In addition to DFO and high-dose baicalein, all other groups showed decreased WBC, Hb, and PLT levels (*P*<0.05 or *P*<0.01) compared with normal values. In comparison with Model controls, only DFO had higher WBC levels (mean 2.24×10^9^/l vs 1.2×10^9^/l in the model,* P*<0.01), which were higher than in all the other treatment groups (*P*<0.05 or *P*<0.01). In addition to low-dose baicalein, all other treatment groups showed increased levels of Hb, and DFO was superior to both low-dose groups (*P*<0.05). Hb for either dose of curcumin was higher than that of low-dose baicalein. Moreover, all treatments showed no effects on PLT recovery. On day 35 (Figure 3), all treatment groups and the Model control group still had lower WBC and PLT levels in comparison with normal values (*P*<0.05 or *P*<0.01). In comparison with untreated Model controls, differences in WBC counts were not observed in all treatment groups. Both curcumin doses and low-dose baicalein significantly increased Hb amounts (*P*<0.05 or *P*<0.01); meanwhile, DFX administration resulted in notably increased PLT amounts (mean 654.8×10^9^/l, vs 396.8×10^9^/L in model, *P*<0.05). High-dose baicalein was superior in elevating PLT levels to low-dose (427.4 and 247.25×10^9^/l, respectively,* P*<0.05). 

**Figure 4:**
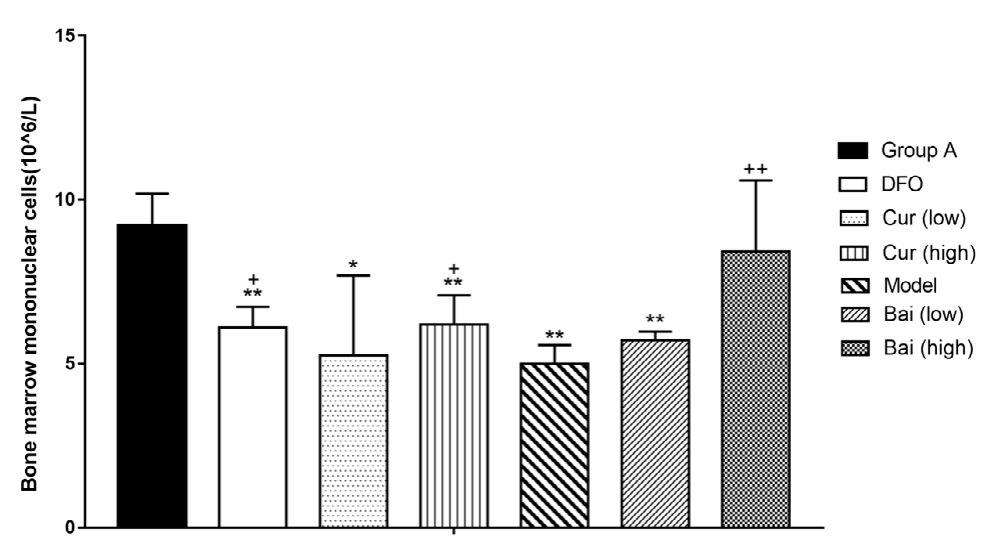
Bone marrow mononuclear cell counts on day 35. Data are mean±SEM (n=8), **P<0.01 (vs. Normal group); +*P<*0.05, ++*P<*0.01 (vs. Model group)

DFO: Deferoxamine; Cur: Curcumin; Bai: Baicalein

Bone marrow mononuclear cells (BMNCs) were also counted manually on day 35. In addition to the high-dose baicalein-treatment group, all the other groups displayed significantly lower levels of BMNCs (*P*<0.05 or *P*<0.01). Administration of DFO and high-dose curcumin or baicalein increased BMNC amounts in comparison with Model controls (*P*<0.05 or *P*<0.01). High-dose curcumin or baicalein led to increased MNCs levels compared with the low-dose groups but with no statistically significant differences (Figure 4). 

**Figure 5:**
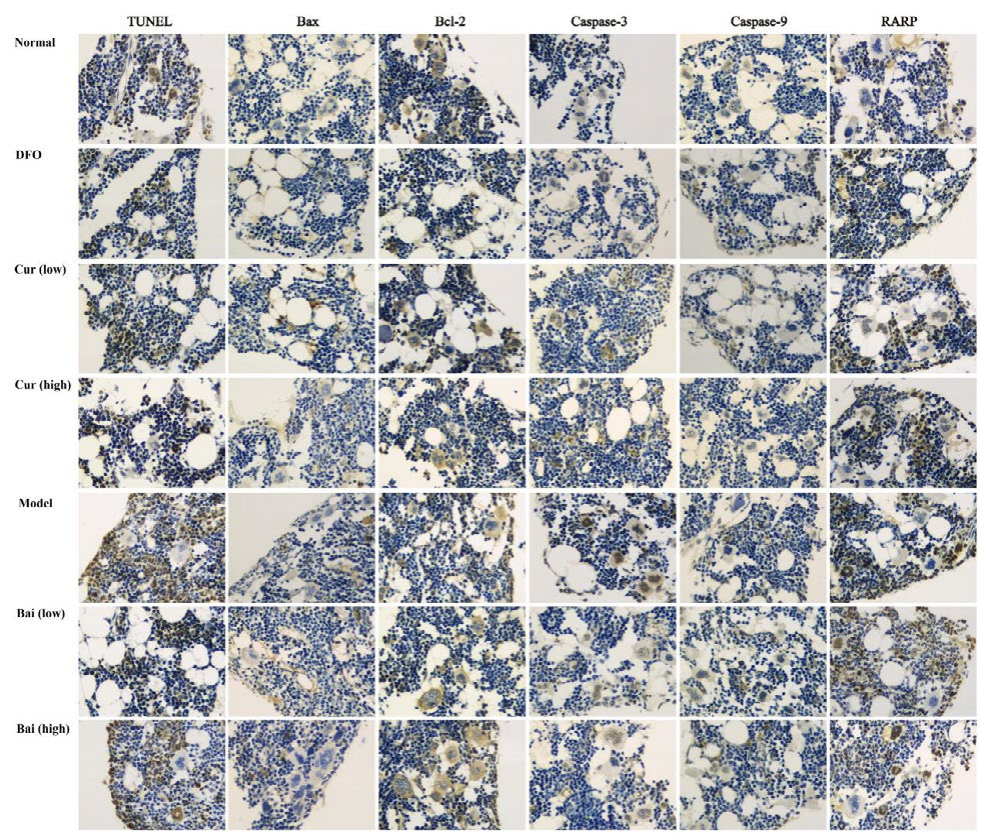
TUNEL assay and expression levels of apoptosis-related proteins in bone marrow samples. After decalcification, fixation, dehydration, paraffin-embedding, and sectioning, TUNEL apoptosis assay and immunohistochemistry were carried out. Analysis was performed by light microscopy at 400×

DFO: Deferoxamine; Cur: Curcumin; Bai: Baicalein; TUNEL: TdT-mediated dUTP-biotin nick end labeling

**Figure 6:**
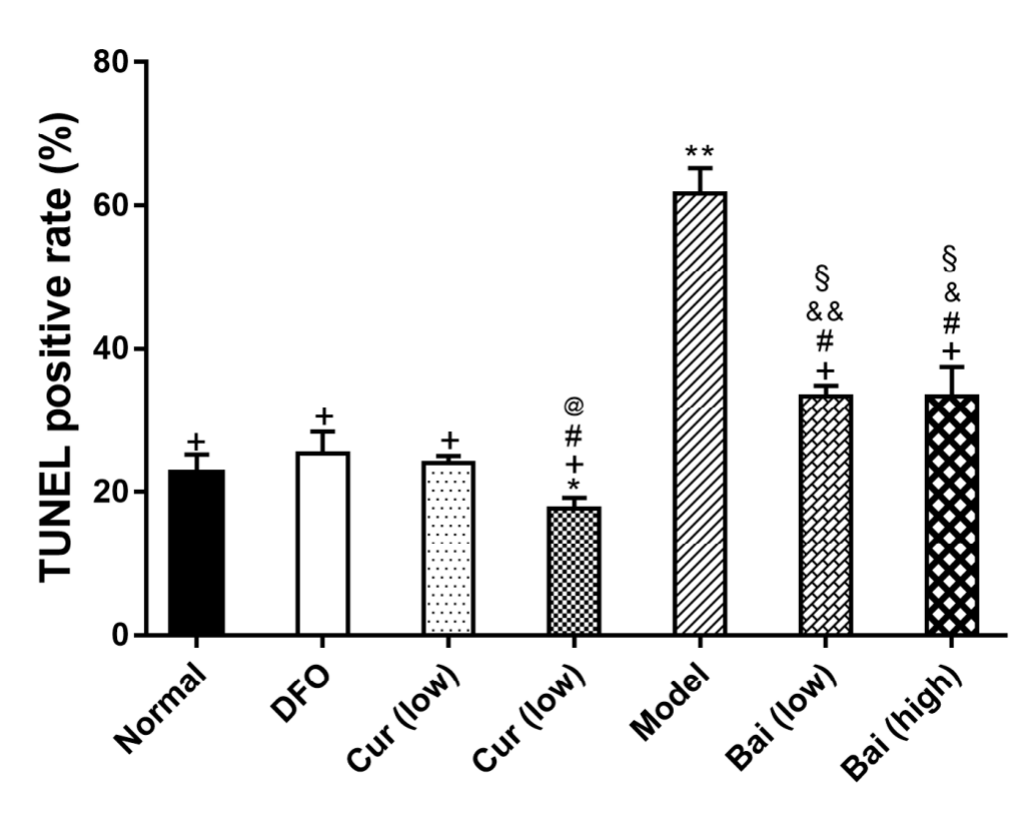
TUNEL positive rates (%) in bone marrow cells. Data are mean±SEM (n=8), *P<0.05, **P<0.01 (vs Normal group); +*P<*0.05, ++*P<*0.01 (vs Model group); #*P<*0.05, ##*P<*0.01 (vs DFO group); @*P<*0.05 (vs corresponding low-dose group). Data are mean±SEM (n=4), *P<0.05, **P<0.01 (vs Normal); +*P<*0.01 (vs Model); #*P<*0.05, ##*P<*0.01 (vs DFO); @*P<*0.01 (vs corresponding low-dose group); & *P<*0.05, && *P<*0.01 (vs low-dose curcumin) and § *P<*0.01 (vs high-dose curcumin)

DFO: Deferoxamine; Cur: Curcumin; Bai: Baicalein; TUNEL: TdT-mediated dUTP-biotin nick end labeling

**Figure 7:**
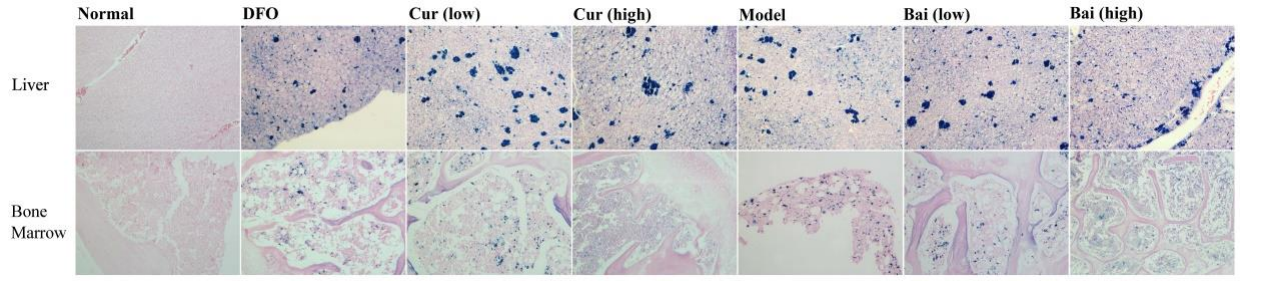
Iron accumulation in the liver and bone marrow. After decalcification (unilateral femur), the samples underwent fixation, dehydration, paraffin-embedding, sectioning, and staining with hematoxylin and eosin/iron (Sudan red). The slides were assessed under a light microscope, with images acquired at a magnification of 400×

DFO: Deferoxamine; Cur: Curcumin; Bai: Baicalein

**Figure 8:**
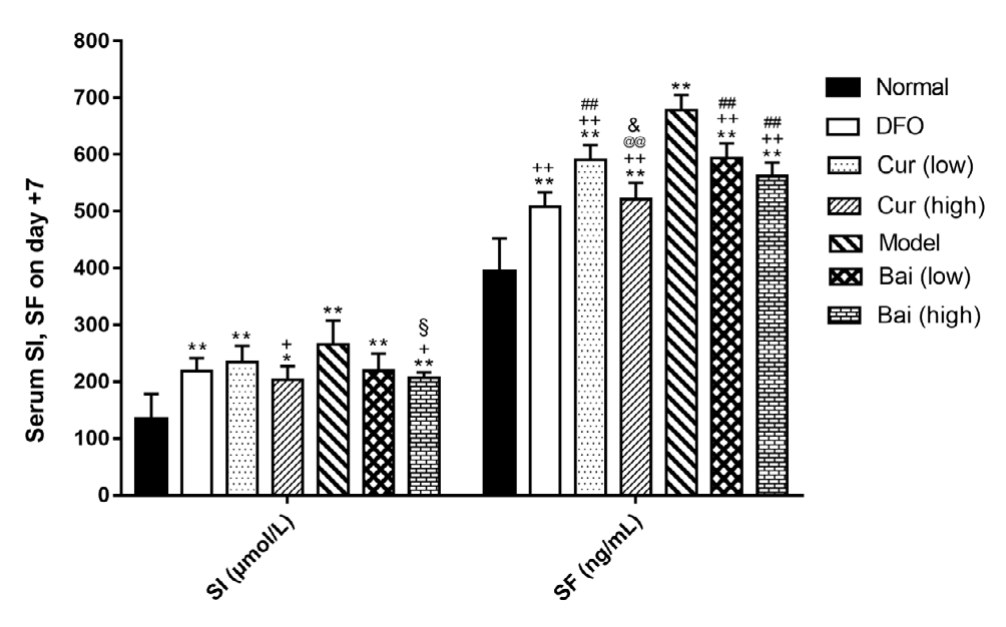
Serum iron and ferritin levels on day 7. Data are mean±SEM (n=4), *P<0.05, **P<0.01 (vs Normal); +*P<*0.01 (vs Model); #*P<*0.05, ##*P<*0.01 (vs DFO); @@*P<*0.01 (vs corresponding low-dose group); &*P<*0.05 (vs same dose group of curcumin and baicalein), and §*P<*0.05 (paired comparison on day 35)

DFO: Deferoxamine; Cur: Curcumin; Bai: Baicalein; SI: serum iron; SF: serum ferritin

**Figure 9:**
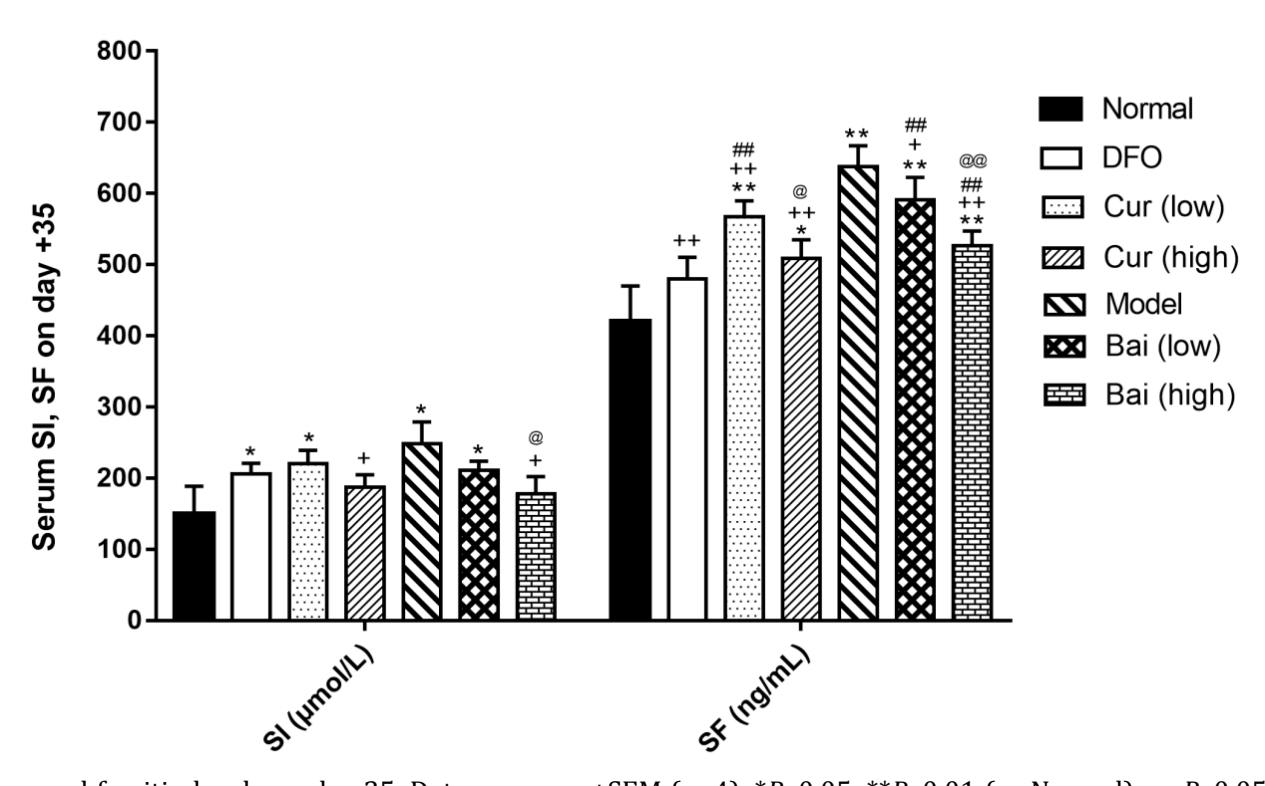
Serum iron and ferritin levels on day 14. Data are mean±SEM (n=4), *P<0.05, **P<0.01 (vs Normal); +*P<*0.01 (vs Model); #*P<*0.05, ##*P<*0.01 (vs DFO); @*P<*0.05 (vs corresponding low-dose group); §*P<*0.05 (paired comparison on day 35)

DFO: Deferoxamine; Cur: Curcumin; Bai: Baicalein; SI: serum iron; SF: serum ferritin

**Figure 10:**
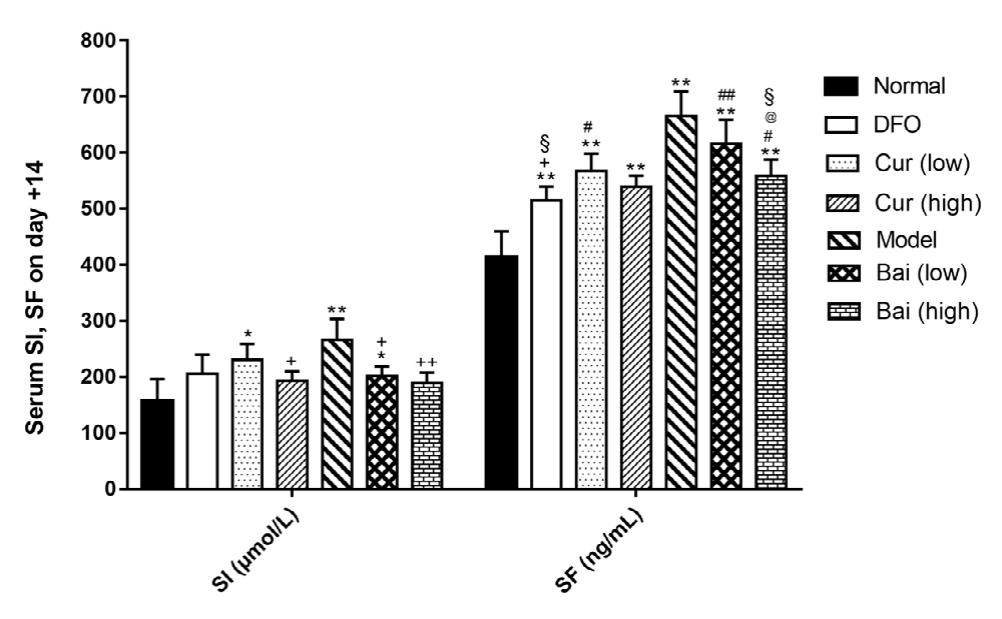
Serum iron and ferritin levels on day 35. Data are mean±SEM (n=4), *P<0.05, **P<0.01 (vs Normal); ++*P<*0.05, +*P<*0.01 (vs Model); ##*P<*0.01 (vs DFO); @*P<*0.05, @@*P<*0.01 (vs corresponding low-dose group)

DFO: Deferoxamine; Cur: Curcumin; Bai: Baicalein; SI: serum iron; SF: serum ferritin

**Figure 11:**
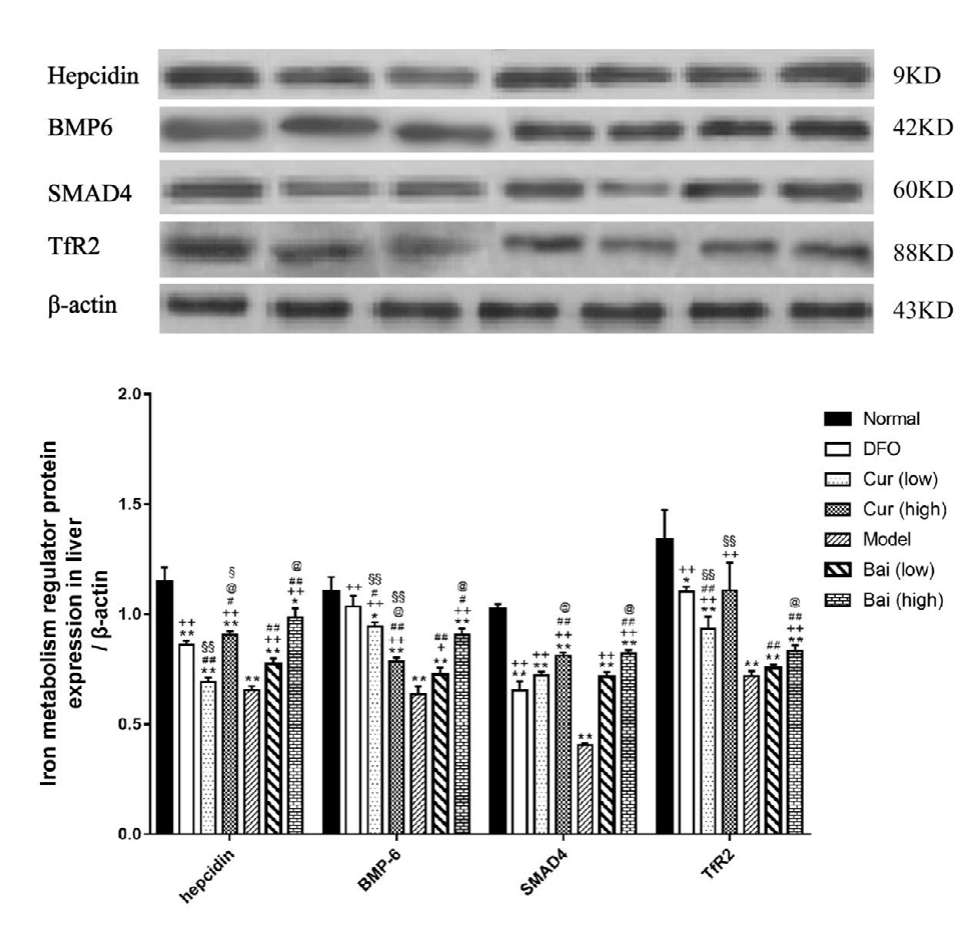
Hepcidin, BMP-6, SMAD4, and TfR2 protein expression levels in the liver on day 35. Data are mean±SEM (n=4), *P<0.05, **P<0.01 (vs Normal); ++*P<*0.05, +*P<*0.01 (vs DFO); #*P<*0.05, ##*P<*0.01 (vs Model); @*P<*0.05, @@*P<*0.01 (vs corresponding low-dose group); §P<0.05, §§P<0.01 (vs. same dose group of curcumin and baicalein)

DFO: Deferoxamine; Cur: Curcumin; Bai: Baicalein; BMP-6: Bone morphogenetic protein 6; SMAD4:SMAD family member4; TfR2: Transferrin receptor 2

**Figure 12:**
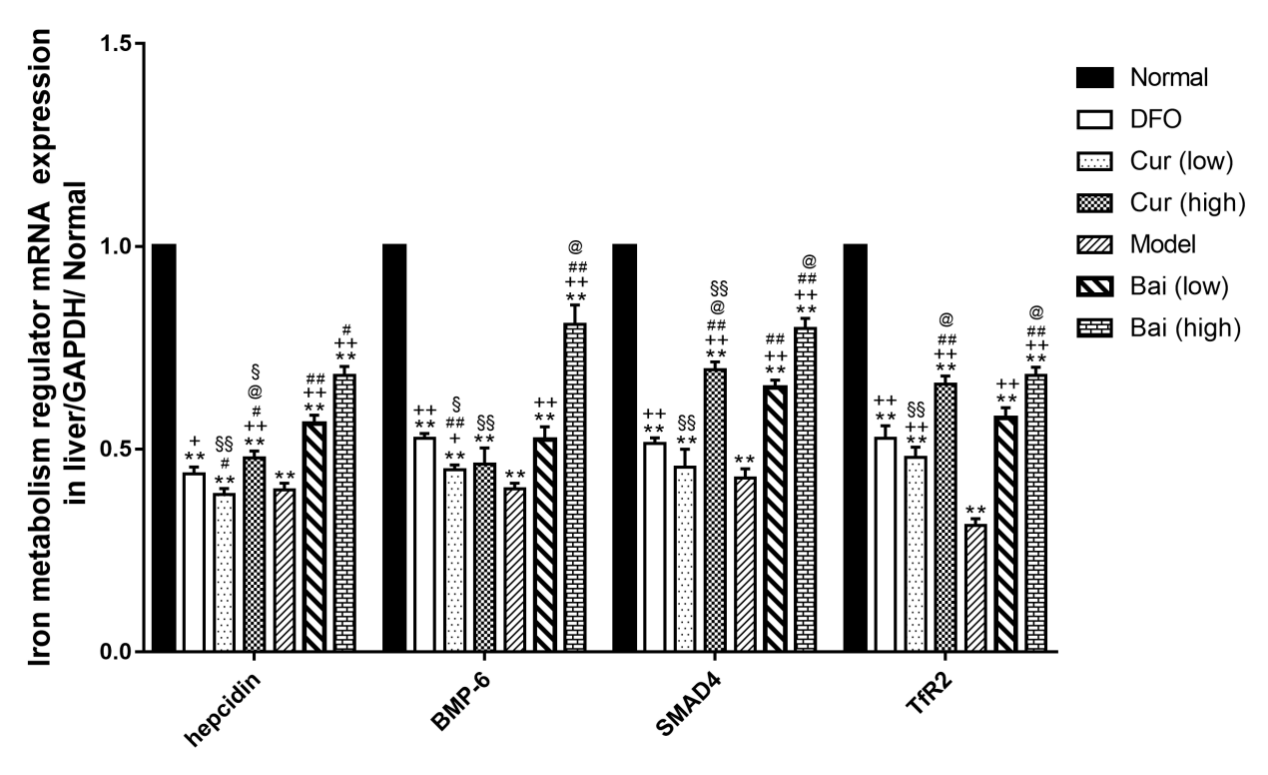
*Hepcidin*, *BMP-6, SMAD4*, and *TfR2* mRNA amounts in the liver on day 35. Data are mean±SEM (n=4), *P<0.05, **P<0.01 (vs Normal); ++*P<*0.05, +*P<*0.01 (vs DFO); #*P<*0.05, ##*P<*0.01 (vs Model); @*P<*0.05 (vs corresponding low-dose group); §*P<*0.05, §§*P<*0.01 (vs same dose group of curcumin and baicalein)

DFO: Deferoxamine; Cur: Curcumin; Bai: Baicalein; BMP-6: Bone morphogenetic protein 6; SMAD4:SMAD family member4; TfR2: Transferrin receptor 2

***Apoptosis in bone marrow and the expression of related protein***

To investigate the mechanisms behind the protective effects on hematopoiesis, apoptosis in bone marrow cells was detected by TUNEL assay and ICH. The TUNEL-positive rate in untreated Model controls was markedly elevated compared with that of the normal group (means of 61.7 and 22.7%, respectively; *P*<0.05). Interestingly, all treatments reduced apoptotic rates (*P*<0.01) (Figure 5 and 6). High-dose curcumin resulted in lower apoptosis rate compared with low-dose (*P*<0.01), and both showed reduced levels compared with either dose of baicalein (*P*<0.05 or *P*<0.01). ICH demonstrated markedly higher expression levels of Bax, caspases 3 and 9, and RARP in untreated model animals compared with the normal group (*P*<0.05). Treatment with DFO, high-dose curcumin, and baicalein reduced the expression levels of all the above indexes, without notable differences compared with the normal group; only caspase-3 was significantly decreased in after treatment with DFO, high-dose curcumin, and both doses of baicalein (*P*<0.05). High-dose curcumin or baicalein increased the expression levels of Bcl-2, albeit without statistically significant differences (Figure 5, Table 2).

***Iron deposition in liver and bone marrow***

During the process of iron overload, the liver and bone marrow are the primary organs involved with heavy iron deposition. Iron deposition in all groups was assessed after H&E/iron staining (Sudan red). The model groups (Groups B–E) had significantly poorer bone marrow hyperplasia than the normal group, with increased amounts of fat granules, reduced hematopoietic cell rates, decreased bone marrow megakaryocyte amounts, as well as marked iron deposition in the bone marrow and liver. After iron chelation treatment for 35 days, hematopoiesis was improved significantly in model animals in comparison with untreated counterparts, manifested by less fat granules and higher hematopoietic cell ratios. These phenomena were rather distinguished in the high-dose curcumin and baicalein groups, and bone marrow iron deposition was decreased significantly in the high-dose curcumin, high-dose baicalein, and DFO groups; the first two groups exhibited optimal results. No significant decrease in liver iron deposition was observed in all treatment groups (Figure 7).

***SI and SF expression levels***

To evaluate iron burden in different groups, serum iron (SI) and serum ferritin (SF) were detected on days 7, 14, and 35, respectively by enzyme-linked immunosorbent assay (ELISA). On day 7 (Figure 8), all model groups showed higher serum SI and SF levels compared with normal values (*P*<0.05); meanwhile, high-dose curcumin or baicalein decreased serum SI (203.4 and 207.5 μmol/l, respectively) in comparison with untreated Model controls (265.3 μmol/l, *P*<0.05). All treatment groups showed significantly reduced SF levels in comparison with untreated models (*P*<0.01); moreover, DFO exhibited the most significant effect (*P*<0.01) that was equivalent to treatment with high-dose curcumin. On day 14 (Figure 9), only the Model control and low-dose curcumin and baicalein treatment groups showed higher SI and SF levels compared with the normal group; high-dose curcumin and either dose of baicalein decreased SI levels compared with the Model control group (*P*<0.05 or *P*<0.01). In addition to DFO treatment (*P*<0.05), all other treatment groups showed no SF level reduction compared with the control group; in the DFO, high-dose curcumin, and low-dose baicalein treatment groups, SF levels were increased compared with those obtained on day 7, but not statistically significant. On day 35 (Figure 10), along with the high-dose curcumin and baicalein groups, all the other groups showed SI levels remaining higher than normal values (*P*<0.05); either high-dose treatment decreased SI levels notably in comparison with untreated models (*P*<0.05). Thus, all treatments could reduce SF levels significantly in comparison with untreated models (*P*<0.05), with DFO exhibiting a significant effect (*P*<0.01) that was similar to that of high-dose curcumin. Either of the high-dose treatment exhibited increased efficiency in reducing SF levels in comparison with low-dose groups (*P*<0.05 or *P*<0.01), and high-dose baicalein also had lower SI levels than the low-dose (*P*<0.05). During the whole iron chelation process, based on the effects exerted on day 7, SI levels were further decreased on day 5 by high-dose curcumin (*P*<0.05), and SF decreased further by DFO and high-dose baicalein (*P*<0.05). 

***Expression levels of iron metabolism regulators***

Hepcidin can negatively modulate iron transportation to the circulation; therefore, we further detected the expression levels of hepcidin and its main regulators, hemojuvelin (HJV) bone morphogenic protein (BMP) Sekelsky Mothers Against DPP (SMAD) signaling pathway, by Western blot and RT-qPCR. The Model control group showed markedly reduced liver hepcidin, bone morphogenetic protein 6 (BMP-6), SMAD family member4 (SMAD4) and transferrin receptor 2 (TfR2) protein amounts (*P*<0.01). Meanwhile, DFO could restore the levels of BMP-6 to normal values, while all the other treatment groups had lower values (*P*<0.05 or *P*<0.01). In addition to hepcidin in the low-dose curcumin group and TfR2 in the low-dose baicalein group, the other treatment groups showed increased hepcidin, BMP-6, SMAD4, and TfR2 protein levels (*P*<0.05). Among these, high-dose curcumin was most effective in elevating hepcidin (*P*<0.01); DFO optimally elevated BMP-6 levels (*P*<0.05), while high-dose curcumin and baicalein enhanced SMAD4 expression similarly (*P*<0.01). The effect of DFO was similar to that of high-dose curcumin in promoting the expression of TfR2 (*P*<0.01). Additionally, high-dose curcumin elevated hepcidin and SMAD4 protein amounts (*P*<0.01) but not BMP-6 in comparison with low-dose; meanwhile, high-dose baicalein was superior to low-dose in elevating the amounts of all the above molecules (*P*<0.01). Baicalein was superior to curcumin with respect to hepcidin level enhancement (*P*<0.05 or *P*<0.01), but inferior in TfR2 expression (*P*<0.01). Also, low-dose curcumin exerted an effect equivalent to high-dose baicalein in promoting BMP-6 expression (*P*<0.01) (Figure 11). RT-qPCR showed that all model groups had reduced *hepcidin*, *BMP-6*, *SMAD4*, and *TfR2 *mRNA levels (*P*<0.01). In addition to *hepcidin *gene expression, *SMAD4* mRNA levels in low-dose curcumin and *BMP-6* mRNA amounts in high-dose curcumin were markedly elevated (*P*<0.01). Of all treatment groups, high-dose baicalein optimally promoted the mRNA expression of *hepcidin*, *BMP-6*, and *SMAD4*, and both high-dose groups were superior in increasing the levels of *TfR2* mRNA to the other groups. DFO only exerted better effects in increasing mRNA levels of hepcidin and *BMP-6* than the low-dose curcumin group (Figure 12). 

## Discussion

Immune-induced bone marrow failure is the main reason for AA development, especially cytotoxic T-cell-mediated hematopoietic stem/progenitor cell apoptosis (26), and patients suffering from iron-overload may present a depressed hematopoiesis and transfusion requirement (6). DFO was the first effective iron-chelator employed in treating AA complicated with iron-overload (9); it served as a positive control in this study. 

The current results indicated that both curcumin and baicalein could improve WBC count and Hb levels after early iron chelation treatment in mice with AA complicated with iron-overload. Subsequently, the effects of high-dose curcumin and baicalein in elevating WBC count or Hb levels were not inferior to those of DFO treatment. Significantly increased PLT amounts were observed only in the DFO treatment group on day 35, which might be an advantage for DFO application. Improvement in peripheral hemogram was in accordance with increased amounts of bone marrow mononuclear cells after iron-chelation treatment, and no significant differences were observed among DFO and high-dose curcumin/baicalein treatment groups. After treatment, we also found that iron chelation treatments remarkably decreased the apoptotic rates of bone marrow cells as assessed by TUNEL assay, especially in the DFO and low-dose curcumin treatment groups. In addition, apoptosis-related protein expression levels were improved as a result of DFO administration as well as high-dose curcumin and baicalein treatments, especially with decreased caspase-3 expression.

In addition to promoting hematopoiesis, iron chelation treatment reduced the iron burden in the model. After H&E/iron staining, iron accumulation in the bone marrow and liver was assessed after 35 days of treatment. The results indicated remarkable iron accumulation in the model groups (bone marrow and liver), and treatment, especially with high-dose curcumin or baicalein, significantly improved hematopoietic cell ratios, decreased fat granule amounts, and eliminated iron deposition in the bone marrow, while the effect of lessening iron deposition in the liver was limited. 

The most convenient and noninvasive method for evaluating the iron burden is the detection of SI and SF levels, which is widely applied in clinical practice (27). In the current mouse model, significantly higher SI and SF levels were observed compared with normal values, and treatment with high-dose baicalein more efficiently reduced SI levels than DFO administration, although the difference was not significant. During treatment, SF levels were not decreased further on day 14 compared to day 7, in accordance with clinical manifestations. Iron may be released from the organ (liver and spleen) into peripheral blood during the process of iron chelation, with increased levels of SF (28). On the other hand, after 35 days of treatment, further decrease was observed of SF levels, which were extremely low in the DFO and high-dose curcumin groups; high-dose baicalein was inferior to DFO in this aspect. 

In iron absorption, iron is firstly transferred by divalent metal transporter 1 from intestinal epithelial cells, with further storage in SF or transfer to plasma by ferroportin (FPN) (29). Hepcidin is considered one of the major negative regulators of iron assimilation, which can induce FPN endocytosis, phosphorylation, and catabolism (30). Several networks regulate hepcidin expression, with a critical role for HJV-BMPSMAD pathway (31). HJV binds type 1 BMP receptor and induces BMP. Meanwhile, SMAD 1/5/8 phosphorylation promotes SMAD4 binding, further generating a complex which is translocated into the nucleus to induce hepcidin expression (32). TfR2 can increase BMP expression, upregulating hepcidin (33), which is strongly expressed in the liver. Furthermore, the above results revealed that hepcidin, BMP-6, SMAD4, and TfR2 amounts at the protein and mRNA levels were significantly lower in untreated model animals. Meanwhile, iron chelation treatment could reverse the suppression of these regulators; specifically, high-dose curcumin was most efficient in elevating hepcidin expression, while DFO maximally increased BMP-6 levels. Additionally, both high-dose curcumin and baicalein improved SMAD4 expression similarly, and DFO displayed an effect similar to that of high-dose curcumin with respect to TfR2 protein expression. Interestingly, high-dose baicalein optimally upregulated BMP-6, hepcidin and SMAD4 at the mRNA level, and both high-dose groups showed increased mRNA levels of *TfR2* compared with the other groups. 

## Conclusion

Both curcumin and baicalein exert effects by upregulating hepcidin and its regulators in the HJV-BMPSMAD pathway. In addition, high-doses of these drugs might be more beneficial than DFO. During hematopoiesis recovery, DFO has an advantage in elevating PLT, and WBC count and Hb level improvements might be similar in the DFO and high-dose treatment groups. Further studies are essential to comprehensively evaluate the safety and mechanisms underlying the effects of curcumin and baicalein in AA in order to elucidate the optimal dosages.
